# Lifestyle intervention before and during fertility treatment in women with obesity or with overweight and PCOS—a RCT

**DOI:** 10.1210/clinem/dgaf660

**Published:** 2025-12-16

**Authors:** Matea Belan, Madélie Giguère-Johnson, Belina Carranza-Mamane, Marie-Hélène Pesant, Youssef AinMelk, Marie-France Langlois, Hélène Lavoie, Ellen M Greenblatt, Sheila Laredo, Margaret Sagle, Guy Waddell, Farrah Jean-Denis, Jean-Patrice Baillargeon

**Affiliations:** Research Center of the Centre hospitalier universitaire de Sherbrooke (CR-CHUS), Sherbrooke, QC J1H 5N4, Canada; Department of Medicine, Division of Endocrinology, Université de Sherbrooke, Sherbrooke, QC J1H 5N4, Canada; Research Center of the Centre hospitalier universitaire de Sherbrooke (CR-CHUS), Sherbrooke, QC J1H 5N4, Canada; Department of Obstetrics and Gynecology, Division of Gynecologic Reproductive Endocrinology and Infertility, Université de Sherbrooke, Sherbrooke, QC J1H 5N4, Canada; Research Center of the Centre hospitalier universitaire de Sherbrooke (CR-CHUS), Sherbrooke, QC J1H 5N4, Canada; Department of Medicine, Division of Endocrinology, Université de Sherbrooke, Sherbrooke, QC J1H 5N4, Canada; Department of Obstetrics and Gynecology, Division of Gynecologic Reproductive Endocrinology and Infertility, Université de Sherbrooke, Sherbrooke, QC J1H 5N4, Canada; Research Center of the Centre hospitalier universitaire de Sherbrooke (CR-CHUS), Sherbrooke, QC J1H 5N4, Canada; Department of Medicine, Division of Endocrinology, Université de Sherbrooke, Sherbrooke, QC J1H 5N4, Canada; Clinic of Medicine and Reproductive Biology, Centre hospitalier de l'Université de Montréal (CHUM), Montréal, QC H2X 0C1, Canada; Mount Sinai Fertility, Mount Sinai Hospital, Toronto, ON M5T 2Z5, Canada; Division of Endocrinology, Michael Garron Hospital, Toronto, ON M4C 3E7, Canada; Department of Obstetrics & Gynecology, University of Alberta, Edmonton, AB T6G 2R3, Canada; Department of Obstetrics and Gynecology, Université de Sherbrooke, Sherbrooke, QC J1H 5N4, Canada; Research Center of the Centre hospitalier universitaire de Sherbrooke (CR-CHUS), Sherbrooke, QC J1H 5N4, Canada; Research Center of the Centre hospitalier universitaire de Sherbrooke (CR-CHUS), Sherbrooke, QC J1H 5N4, Canada; Department of Medicine, Division of Endocrinology, Université de Sherbrooke, Sherbrooke, QC J1H 5N4, Canada

**Keywords:** obesity, fertility, women, lifestyle intervention, live-birth, randomized controlled trial

## Abstract

**Context:**

Lifestyle interventions are recommended for women with obesity and infertility, but trial evidence is scarce, and studies have not investigated interventions that continue during fertility treatment.

**Objective:**

Evaluate effectiveness of a lifestyle intervention compared with usual care on fertility outcomes in women with obesity and infertility.

**Design:**

The Obesity-Fertility randomized controlled trial (RCT), at an academic fertility clinic, included 127 women aged 18 to 40 years with infertility and obesity (body mass index ≥30 kg/m^2^, or ≥27 kg/m^2^ with polycystic ovary syndrome [PCOS]), excluding those unlikely to conceive naturally. The intervention group (IG) received a 6-month lifestyle intervention alone (individual follow-up with a dietician and kinesiologist, and group sessions) before adding fertility treatments. The control group (CG) received usual fertility care directly. Rate of live birth conceived within 18 months of randomization was analyzed.

**Results:**

Compared to CG, the IG lost more weight (−3.21% ± 4.73% vs −0.40% ± 3.66%, *P* = .003) and waist circumference (−2.62 ± 4.46 cm vs −0.23 ± 3.81 cm, *P =* .01) at 6 months. The live-birth rate was 44.4% in the IG (n = 63) vs 35.9% in the CG (n = 64) (risk ratio [RR] = 1.24 [95% CI 0.81-1.90]). In IG vs CG, clinical pregnancy rates were 52.4% vs 37.5% (RR = 1.40 [0.94-2.07]) and natural pregnancy rates were 27.0% vs 12.5% (RR = 2.16 [1.01-4.64]).

**Conclusion:**

Compared with usual care, a lifestyle intervention alone for 6 months and then combined with fertility treatments did not increase the 18-month rate of pregnancies leading to a live birth, but significantly increased natural pregnancy rates, potentially reducing the need for costly assisted reproduction in this population.

Approximately 10% to 15% of Canadian couples experience infertility (1). The most common cause of infertility in women with anovulation is polycystic ovary syndrome (PCOS), which affects 1 in 10 women of reproductive age (20 to 44 years old) (2). Obesity, defined as a body mass index (BMI) ≥30.0 kg/m^2^) (3), contributes to the development and clinical manifestations of PCOS (4) but has also been associated with reduced fertility even in ovulating women (5-8). Indeed, a study involving 3029 subfertile couples with ovulatory cycles, patent tubes, and normal sperm counts found that there was a 4% decrease in conception rate for every 1 kg/m^2^ increase in BMI between 30 and 35 (8). In Canada, as of 2015, 24% and 22% of women aged 18 to 44 years had a measured BMI of 25 to 29.9 kg/m^2^ and ≥30.0 kg/m^2^, respectively (9). In addition, many studies found that mothers with prepregnancy obesity displayed higher incidence of adverse outcomes during pregnancy, including gestational diabetes, preeclampsia, cesarean deliveries, and intrauterine death (10-12). Moreover, their offspring were at higher risk for early development of obesity, type 2 diabetes, and cardiovascular disease (13, 14).

To prevent these adverse effects of obesity on female fertility and subsequent pregnancy, many guidelines have recommended, as a first-line treatment, that women with obesity should be assisted, before conception, to lose 5% to 10% of their initial weight (15, 16). Furthermore, studies have shown that adopting and maintaining a healthy lifestyle can improve the reproductive function (17, 18) and decrease risk of adverse pregnancy outcomes in women with obesity (8, 19, 20). Only 2 published randomized controlled trials (RCTs) evaluated a lifestyle intervention in a general population of women with obesity and subfertility, that is, not specific to PCOS or in vitro fertilization (IVF), as compared with usual care (21, 22). The largest and most recent RCT tested a 6-month program before fertility treatment compared with immediate initiation of fertility treatment in 564 women (21). This trial found a higher 24-month cumulative incidence of natural pregnancy in the lifestyle group (26.1% vs 16.2%; risk ratio [RR] 1.61, 95% CI 1.16-2.24) but with a significant decrease in live-birth rate.

Structured lifestyle programs are not part of routine fertility care nor integrated into most fertility clinics, in part because there is still a lack of RCTs assessing the effectiveness of lifestyle programs in women with obesity and infertility (18). Accordingly, the main objective of this study was to determine the impact of the Obesity-Fertility lifestyle program (23) on fertility outcomes of women with obesity and subfertility seeking care at a fertility clinic. The hypothesis of this trial is that a lifestyle intervention in women with obesity and subfertility will improve their live-birth rates compared to usual fertility care.

## Methods

We conducted a pragmatic RCT at the fertility clinic of the Centre hospitalier universitaire de Sherbrooke (CHUS). A detailed protocol of the study was previously published (23).

### Participants

Women seen at the fertility clinic were eligible if they were infertile, aged between 18 and 40 years, and with a BMI ≥30 kg/m^2^ or ≥27 kg/m^2^ for women with PCOS. A lower BMI threshold was used for women with PCOS, as they display metabolic consequences at a lower BMI than women without PCOS and are more likely to benefit from lifestyle changes (24, 25). Women were considered “infertile” if they had criteria for the definition of infertility or were eligible for fertility care, namely because of a failure to achieve a clinical pregnancy: (1) after 1 year or more of regular and unprotected sexual intercourse (definition of infertility) for women under 35 years of age; (2) after 6 months for (i) women between 35 and 40 years of age or (ii) irregular menstrual cycles; or (3) a previously known cause of infertility (23). We excluded women if a natural conception was impossible or highly unlikely (both tubes blocked, partner's total motile sperm count <5 million (severe male factor), recommended for direct IVF or insemination with donor, etc), or if they had undergone or were planning to undergo bariatric surgery.

After the research team obtained written informed consent from participants, eligible patients were randomized 1:1 (by blocks varying from 2 to 6) to 1 of 2 research arms, with stratification for PCOS status. PCOS was defined by a diagnosis stated in the medical record or when criteria of PCOS were present in the medical record based on Rotterdam definition (26). Most women underwent an ovarian ultrasound as part of their fertility workup. Blood samples were assayed for androgens and exclusion criteria by the local hospital laboratories using the following methods: thyroid-stimulating hormone (TSH, Roche Cat# 11731459, RRID: AB_ 2756377) and prolactin (PRL, Roche Cat# 03203093 190, RRID: AB_2883976) were measured by electrochemiluminescence (ECL) sandwich immunoassay on a Modular ECL analyzer (Roche Diagnostics, Mannheim, Germany); total testosterone and 17-hydroxyprogesterone by liquid chromatography–tandem mass spectrometry (LC-MS/MS) on a 6460 triple quadrupole mass spectrometer (Agilent Technologies, Santa Clara, CA, USA); dehydroepiandrosterone sulfate (DHEAS, Siemens Cat# L2KDS2, RRID: AB_2895591) and androstenedione (Siemens Cat# LKAO1, RRID:AB_2895713) by competitive chemiluminescent enzyme immunoassay on the Immulite 2000 analyzer (Siemens Healthcare Diagnostics, Erlangen, Germany); and sex hormone-binding globulin (SHBG, Siemens Cat# L2KSH2, RRID: AB_2819251) by two-site chemiluminescent immunometric assay on the Immulite 2000 analyzer (Siemens Healthcare Diagnostics, Erlangen, Germany).

The group allocation list was generated by an independent statistician, and successive allocations were placed in concealed envelopes. Owing to the nature of the intervention and the organization of the research team, it was not possible to blind participants, those delivering the intervention, or those responsible for data collection and analysis.

### Interventions

The intervention group received an ad hoc 6-month lifestyle intervention followed by fertility treatments starting after 6 months in combination with the lifestyle intervention, while the control group started fertility treatments immediately. The intervention group received individual counseling with a registered dietitian and a kinesiologist at 3 weeks post-randomization and then every 6 weeks (see Duval et al (23) for more details). These professionals used motivational communication techniques (27) and SMART (Specific, Measurable, Attainable, Realistic and Timely) objectives (28) to help participants make progressive and sustainable lifestyle changes. Lifestyle goals were largely individualized, based on the 2007 Canada's Food Guide (29) and with the aim to gradually increase participants' level of physical activity. The intervention also included 12 different educational group sessions comprising a 45-minute interactive workshop on topics relevant to obesity management, fertility, and lifestyle habits; and a 45-minute physical activity session (23). After the first 6 months, nonpregnant participants could begin fertility treatments in parallel to the lifestyle intervention for up to 18 months of participation or the end of a pregnancy.

The control group had immediate access to usual fertility treatment, which was offered to participants in both groups according to the recommendations of their fertility specialist. Fertility treatments were individualized according to participants' conditions (eg, PCOS) and preferences, without the use of a standardized fertility treatment protocol imposed on clinicians by the project. Based on clinical standards in this population (30), the usual practice in our center for women with subfertility and eligible for this trial is to perform ovulation induction treatments for 3 to 6 cycles, then, if not pregnant, add intrauterine insemination (IUI) for 3 to 6 cycles, and then go on to an IVF protocol with usually ≤3 frozen embryo transfers. Overall, most women who persevere will have completed their fertility treatments in less than 12 months, even if successful pregnancy is not achieved. Standard fertility treatment may include lifestyle counseling by the patient's fertility specialist.

In our center, IUI is not usually offered to women with a BMI >45 kg/m^2^, and IVF is not offered if the BMI is >40, and is restricted to metabolically stable women with accessible ovaries if the BMI is between 35 and 40. There are no other specific restrictions based on BMI.

The lifestyle intervention in this study was provided free of charge to participants, but the research did not cover any costs related to fertility care, which at the time of the study was mostly covered by provincial public insurance.

### Outcomes

Both groups were followed for 18 months or until the end of a pregnancy confirmed within 18 months of randomization (for live birth or miscarriage). Data collection was scheduled every 6 months, or as early as possible at the onset of a pregnancy and between 24 and 28 weeks of gestation. Medical records of participants and newborns were reviewed after the end of participation to document clinical outcomes.

The primary outcome measure was the rate of live births resulting from a pregnancy that was confirmed during the 18-month period after randomization. Secondary outcomes were categorized as follows: (1) fertility outcomes: clinical pregnancy (confirmed fetal heart at ultrasound or pregnancy at more than 12 weeks of gestation), miscarriage (loss of a clinical pregnancy), and stillbirth (in utero death of a fetus before 28 weeks of gestation (31)); (2) methods of conception for confirmed pregnancy: (i) without fertility treatments (natural), (ii) with ovulation induction with or without intrauterine insemination (IUI), (iii) with IVF, with or without intracytoplasmic sperm injection (ICSI), or frozen embryo transfer; (3) number of fertility treatment cycles; and (4) anthropometric measures: weight, fat mass, and waist circumference. Note that women who had a miscarriage were allowed to continue in the study and could have another pregnancy during follow-up, possibly with a life birth. In this case, the participant would be considered to have had a clinical pregnancy, a miscarriage, and a live birth.

### Sample size

At the time the trial was planned (in 2011), the best available evidence was an Australian RCT showing in 171 women with obesity and subfertility that the Fertility Fitness lifestyle program increased the 18-month pregnancy rates from 21% to 61% *(P <* .001) (Clark AM et al, personal communication, unpublished). So, we calculated that a sample size of 58 women per group would provide our study a power of 80% to identify a doubling in the primary outcome rates (live birth) with our program (from 25% to 50%, two-sided α of 5%). Assuming a drop-out rate of 10%, our final estimated sample size was 128 women.

### Statistical analyses

Primary analyses were planned based on the intention-to-treat (ITT) principle. Differences between groups were compared using risk ratios (RR) with their 95% CI as well as chi-square tests for frequencies, Student's *t* tests for normally distributed and Mann-Whitney tests for non-normally distributed continuous variables. Note that all pregnancy outcomes were known for all participants, so no censoring was required for these analyses. As recommended for pragmatic trials (32), we performed modified per protocol (mPP) analyses excluding women who dropped out in the first 6 months of participation without getting pregnant but keeping those who dropped out after 6 months to limit the extend of potential biases. Between-group comparisons were also adjusted using multivariate models for potential confounding variables that appeared to differ between groups at baseline, namely smoking and PCOS status, parity, BMI categories, and male or tubal factor. Anthropometric changes at 6 months were assessed using data from the participant's regular research visit at 6 months (V6) or first pregnancy research visit (PV1) taking place between 5 to 7 months of follow-up.

The cumulative rate of the primary outcome (pregnancies resulting in a live birth) and of the main secondary outcome (clinical pregnancies) were compared between groups using Kaplan-Meier analyses and log-rank tests. We included censoring for data at 18 months for the participants not achieving the outcomes and, for mPP analyses, at the time they were lost to follow-up for those who dropped out of the study. Analyses were performed using Stata version 17 (descriptive statistics and group comparisons) and SPSS version 28 (survival analyses).

### Ethics approval

This study was approved by the Institutional Research Ethics Review Board of the Centre hospitalier universitaire de Sherbrooke (November 23, 2011; 11-128; CHUS), Quebec, Canada. The study is registered with ClinicalTrials.gov (December 1, 2011; NCT01483612) and its protocol was published in *BMC Obesity* (23). All included participants provided written informed consent prior to enrollment.

## Results

**Figure 1:**
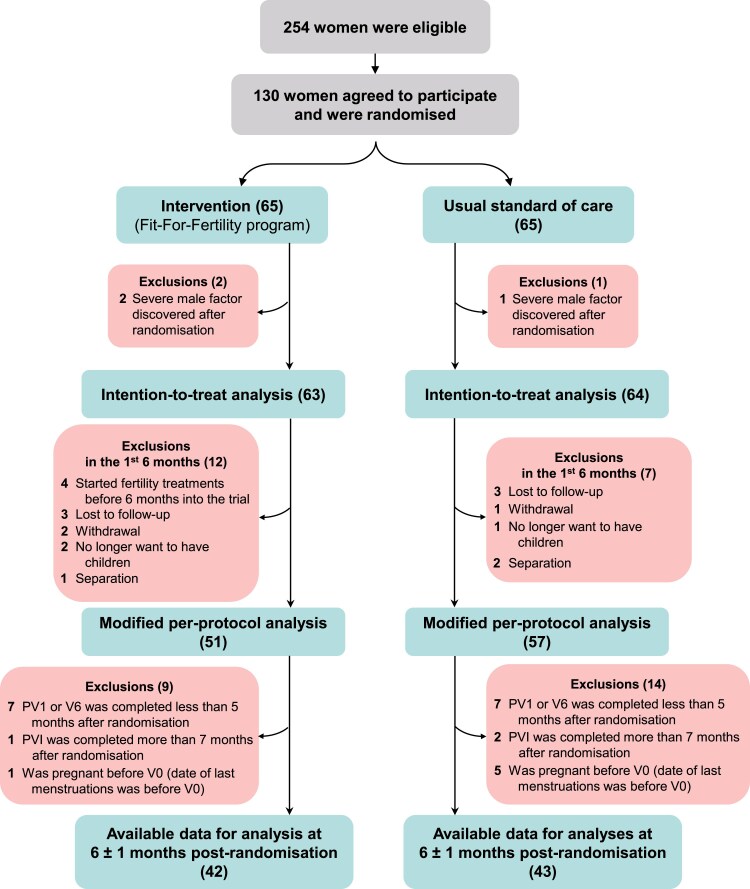
Flow diagram of participants during the trial. Intention-to-treat analysis included 127 women (63 intervention and 64 control). The modified per protocol analysis included only women who have completed at least 6 months into the study or became pregnant during the first 6 months of participation. Abbreviations: PV1: first pregnancy research visit; V0: First research visit; V6: Research visit at 6 months after randomization.

From November 23, 2011, to September 21, 2016, out of 254 interested women referred to the study, 130 were confirmed eligible after the first research visit and agreed to participate. They were then randomized to the control group (n = 65) or the intervention group (n = 65). Three women were excluded afterward because of severe male factor infertility discovered after randomization, resulting in 127 women included in ITT analyses (intervention: 63, control: 64). Note that pregnancy outcomes were available for all participants, even for those who dropped out. After 6 months of follow-up, 12 women in the intervention group and 7 in the control group had dropped out of the study, such that 108 women (85.0% of participants) were included in the mPP analyses (intervention: 51 (81.0%), control: 57 (89.1%)). Figure 1 shows the flow of participants in the trial. Baseline characteristics of the 97 male partners who accepted to participate to a substudy have been previously published (33).

### Anthropometric changes

A total of 42 women in the intervention group and 43 women in the control group had available data at 6 ± 1 months of follow-up and were included in anthropometric analyses (Fig. 1). The intervention group showed significant improvements compared to the control group (data not shown): greater weight loss (−3.14 [4.93] kg vs −0.40 [3.66] kg, *P =* .01), percentage weight loss (−3.21% [±4.73%] vs −0.40% [±3.66%], *P* = .003), waist circumference loss (−2.62 [±4.46] cm vs −0.23 [±3.81] cm, *P =* .01), decrease in fat percentage (1.05% [0.34%] vs 0.49% [±0.19%], *P* = .14), and fat mass (−2.32 [0.67] kg vs −0.64 [0.41] kg, *P =* .04). Additionally, a higher percentage of participants in the intervention group achieved at least 3% (47.6% vs 20.9%, *P* = .01) and 5% weight loss (26.9% vs 9.3%, *P =* .04).

### Intention-to-treat analyses

As shown in Table 1, participants randomized to the 2 arms of the trial did not display clinically important differences at baseline, except perhaps for fewer women who were current smokers or had a BMI ≥ 35 km/m^2^ in the intervention group (differences not statistically significant).

**Table 1:** Baseline characteristics according to trial group for women included in the intention-to-treat analyses

Characteristics (127)	Intervention group (63)	Control group (64)
**Women's characteristics**		
**Age, years**	30.0 (5.2)	29.3 (4.5)
**Education level, n (%)**		
Primary school	0 (0%)	3 (4.7%)
Secondary education	16 (25.4%)	12 (18.8%)
Intermediate vocational education	33 (52.4%)	33 (51.6%)
Advanced vocational education or university	14 (22.2%)	11 (17.2%)
**Current smoker, n (%)**	13 (20.6%)	19 (29.7%)
**Nulliparous, n (%)**	33 (52.4%)	43 (67.2%)
**BMI, km/m^2^**	39.4 (0.96)	40.0 (0.95)
27 ≥ BMI ≤ 30 km/m^2^	7 (11.1)	5 (7.8)
30 > BMI ≤ 35 km/m^2^	19 (30.2)	14 (21.9)
BMI ≥ 35 km/m^2^	37 (58.7)	45 (70.3)
**Waist circumference, cm**	115.0 (13.9)	115.5 (16.0)
**Infertility diagnosis, n (%)**		
Anovulatory	47 (74.6%)	50 (78.1%)
PCOS	42 (66.7%)	39 (60.9%)
Male factor	4 (6.4%)	6 (9.4%)
Tubal factor (Fallopian tube blocked)	4 (6.4%)	7 (10.9%)
Other or unknown cause of infertility	7 (11.1%)	9 (14.1%)

Results are reported as mean (SD) or frequencies (with percentage).

Abbreviations: BMI, body mass index; and PCOS, polycystic ovary syndrome (based on medical record or Rotterdam criteria (24)).

### Fertility outcomes

The lifestyle intervention did not significantly improve the primary outcome rate—live birth after conception occurring within 18 months following randomization—in comparison to the control group (risk ratio = 1.24 (95% CI: 0.81 to 1.90), *P* = .33) (Table 2). The absolute percentage increase was 8.5% (95% CI: −8.5% to +25.5%), which means that if this difference were to be confirmed in a larger study with adequate statistical power, 12 women would need to be enrolled in our lifestyle program to achieve one additional live birth (ie, number needed to treat [NNT], with 95% CI crossing zero). Although not statistically significant, there was a higher clinical pregnancy rate in the intervention group vs the control group, with an absolute percentage increase of +14.9% (95% CI: −2.2% to +32.0%; (*P* = .09). Miscarriage and stillbirth rates did not differ between groups. Notably, 1 multiple gestation occurred in the intervention group following ovulation induction with IUI, and 1 in the control group after ovulation induction alone.

**Table 2:** Pregnancy outcomes within 18 months after randomization according to group, for women included in the intention-to-treat analyses

Outcomes	Intervention group (n = 63)	Control group (n = 64)	Risk ratio (95% CI)
**Primary outcome: live birth**	28 (44.4%)	23 (35.9%)	1.24 (0.81 to 1.90)
**Clinical pregnancy**	33 (52.4%)	24 (37.5%)	1.40 (0.94 to 2.07)
**Miscarriage (total number of miscarriages)**	7 (11.1%)	5 (7.8%)	1.42 (0.48 to 4.25)
**Miscarriage (total number of women)**	6 (9.5%)	5 (7.8%)	1.22 (0.39 to 3.79)
**Multiple gestation (twins)**	1 (1.6%)	1 (1.6%)	1.02 (0.07 to 15.9)
**Stillbirth**	0	0	N/A

Definitions: Live birth: baby delivered after 37 weeks of gestation from a pregnancy occurring within 18 months after randomization; Clinical pregnancy: confirmed fetal heart at ultrasound or pregnancy at more than 12 weeks of gestation; Miscarriage: loss of a clinical pregnancy; and Stillbirth: intrauterine death of a fetus after 28 weeks of gestation.

**Figure 2:**
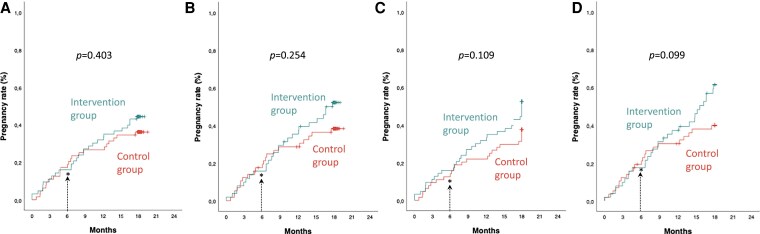
Time to pregnancy. Kaplan-Meier curves showing the time to pregnancy resulting in a live birth (A and B) or independently of the end result (C and D), according to intention-to-treat (A and C) and modified per protocol analyses (B and D). Green line (on top) = intervention group. Red line (below) = control group. *End of the first 6 months of participation and addition of fertility treatments to the lifestyle program for the intervention group. Panel A: Pregnancy resulting in a live birth, intention-to-treat analysis. Panel B: Pregnancy resulting in a live birth, modified per protocol analysis. Panel C: Clinical pregnancy (including miscarriage and live birth), intention-to-treat analysis. Panel D: Clinical pregnancy (including miscarriage and live birth), modified per protocol analysis.

Kaplan-Meier curves showed no significant difference between groups for pregnancy resulting in a live birth (Fig. 2A; log rank *P* = .41) or clinical pregnancy (Fig. 2C; log rank *P =* .17). The mean time to pregnancy resulting in a live birth was 14.2 months (95% CI: 12.6 to 15.8) for the intervention group and 15.0 months (13.3 to 16.6) for the control group, and respectively 13.6 months (12.0 to 15.2) and 14.3 months (12.7 to 15.9) for the mean time to a clinical pregnancy.

### Methods of conception and fertility treatments

A higher proportion of participants in the intervention group conceived without fertility treatments compared with the control group (RR = 2.16 (1.01 to 4.64), *P* = .049) (Table 3), with an absolute risk increase of +14.5% and an NNT of 7 (95% CI: 4 to 115). This increase remained significant after correction for potential confounding variables (see “Methods”) (*P* = .025). This improvement in natural pregnancies occurred even though there were no differences between the groups in the percentage of participants who did not undergo fertility treatment (33.3% in the intervention group vs 35.9% in the control group; Table 3).

**Table 3:** Methods of conception and outcomes of fertility treatments 18 months after randomization, for women included in the intention-to-treat analyses

Outcome	Intervention group (n = 63)	Control group (n = 64)	Risk ratio (95% CI)
**Method of conception leading to clinical pregnancy (percentage of women)**			
Natural	17 (27.0%)	8 (12.5%)	2.16 (1.01 to 4.64)*^a^*
Ovulation induction, without IUI	6 (9.5%)	9 (14.1%)	0.68 (0.26 to 1.79)
Intrauterine insemination	9 (14.3%)	3 (4.7%)	3.05 (0.87 to 10.7)
IVF (with or without ICSI) or FET	1 (1.6%)	4 (6.3%)	0.25 (0.03 to 2.21)
**Fertility treatments (percentage of women)**			
Any fertility treatment	42 (66.7%)	41 (64.1%)	1.04 (0.81 to 1.34)
Ovulation induction	42 (66.7%)	38 (59.4%)	1.12 (0.86 to 1.47)
Intrauterine insemination	26 (41.3%)	18 (28.1%)	1.47 (0.90 to 2.40)
IVF (with or without ICSI) or FET	4 (6.4%)	4 (6.3%)	1.02 (0.27 to 3.89)
**Fertility treatments (number of cycles)**			
Ovulation induction	163	159	N/A
Intrauterine insemination	73	60	N/A
IVF (with or without ICSI) or FET	4	9	N/A
**Cycles per woman*^b^***			
Ovulation induction	2 [0-5]	2 [0-4]	*P* value = .639
Intrauterine insemination	0 [0-3]	0 [0-1]	*P* value = .167
IVF (with or without ICSI) or FET	0 [0-0]	0 [0-0]	*P* value = .945

Results are reported as frequencies (with percentages).

Abbreviations: FET, frozen embryo transfer; ICSI, intracytoplasmic sperm injection; IUI, intrauterine insemination; IVF, in vitro fertilization.

^*a*^ *P* = .025 after correction for smoking status, PCOS status, parity, BMI categories, and male or tubal factor.

^*b*^Non-normally distributed variables are presented as medians [with interquartile range] and *P* values from Mann-Whitney U test.

There were no statistically significant differences between groups in the rate of clinical pregnancy after different fertility treatment methods, but the intervention group seemed to have higher rates of clinical pregnancy after IUI compared with the control group (*P* = .08). There were no noticeable differences between the 2 groups in the mean number of cycles per woman for each fertility treatment (Table 3). There were no reported complications following fertility treatments in either group.

### Modified per protocol analysis

A total 108 women were included in the mPP analyses. Baseline characteristics show no statistical differences between both groups at baseline (Table 4), although there appear to be fewer current smokers, women with grade 2 obesity (BMI ≥35 kg/m^2^), and male factor infertility in the intervention group.

**Table 4:** Baseline characteristics according to group trial for women included in the modified per protocol analyses

Characteristics (108)	Intervention group (51)	Control group (57)
**Women's characteristics**		
** Age, years**	30.6 (5.0)	29.6 (4.3)
**Education level, n (%)**		
Primary school	0 (0%)	5 (8.77%)
Secondary education	11 (21.6%)	7 (12.3%)
Intermediate vocational education	27 (52.9%)	34 (59.7%)
Advanced vocational education or university	13 (25.5%)	11 (19.3%)
**Current smoker, n (%)**	8 (15.7%)	18 (31.6%)
**Nulliparous, n (%)**	26 (51.0%)	39 (68.5%)
**BMI, km/m^2^**	38.8 (7.3)	40.5 (7.8)
27 ≥ BMI ≤ 30 km/m^2^	6 (11.8%)	4 (7.0%)
30 > BMI ≤ 35 km/m^2^	16 (31.4%)	12 (21.1%)
BMI ≥ 35 km/m^2^	29 (56.9%)	41 (71.9%)
**Waist circumference, cm**	114.2 (13.2)	116.5 (16.3)
**Infertility diagnosis, n (%)**		
Anovulatory	39 (76.5%)	45 (79.0%)
PCOS	33 (64.7%)	35 (61.4%)
Male factor	1 (2.0%)	6 (10.5%)
Tubal factor (one Fallopian tubes blocked)	4 (7.88%)	7 (12.3%)
Other or unknown cause of infertility	6 (11.8%)	7 (12.3%)

Results are reported as mean (SD) or frequencies (with percentage).

Abbreviations: BMI, body mass index; PCOS, polycystic ovary syndrome (based on medical record or Rotterdam criteria (24))

### Fertility outcomes

Although not significant, there was a higher live-birth rate in the intervention group compared to the control group (RR = 1.38 [0.90 to 2.13], *P* = .10) (Table 5), with an absolute percentage increase of +14.2% (95% CI: −4.4% to 32.8%), which would result in an NNT of 7 (95% CI crossing zero) if this difference were to be confirmed in an adequately powered study. The rate of clinical pregnancies was 1.6 times higher in the intervention group than in the control group (*P* = .024), with an absolute percentage increase of +22.2%, which would result in an NNT of 5 (95% CI, 2 to 26). This increase remained statistically significant after adjustment for potential confounding variables (see “Methods” section, *P* = .025). There were no significant differences in miscarriage or stillbirth rates between the groups. In the mPP analysis, 1 multiple gestation was observed in the intervention group (2.0%).

**Table 5:** Pregnancy outcomes within 18 months after randomization according to group, for women included in the modified per protocol analyses

Outcomes	Intervention group (n = 51)	Control group (n = 57)	Risk ratio (95% CI)
**Primary outcome: live birth**	26 (51.0%)	21 (36.8%)	1.38 (0.90 to 2.13)
**Clinical pregnancy**	31 (60.8%)	22 (38.6%)	1.58 (1.06 to 2.34)^*a*^
**Miscarriage (total number of miscarriages)**	7 (13.7%)	4 (7.0%)	1.96 (0.61 to 6.30)
**Miscarriage (total number of women)**	6 (11.8%)	4 (7.0%)	1.68 (0.50 to 5.61)
**Multiple gestation (twins)**	1 (2.0%)	1 (1.75%)	1.12 (0.07 to 17.4)
**Stillbirth**	0	0	N/A

Definitions: Live birth: baby delivered after 37 weeks of gestation from a pregnancy occurring within 18 months after randomization; Clinical pregnancy: confirmed fetal heart at ultrasound or pregnancy at more than 12 weeks of gestation; Miscarriage: loss of a clinical pregnancy; and Stillbirth: intrauterine death of a fetus after 28 weeks of gestation.

^*a*^ *P* = .025 after correction for smoking status, PCOS status, parity, BMI categories, and male or tubal factor.

The survival analyses showed no significant difference between groups for Kaplan-Meier curves of a pregnancy resulting in a live birth (Fig. 2B; log rank *P* = .26) nor for a clinical pregnancy (Fig. 2D; log rank *P =* .08). The mean (95% CI) time to the confirmation of a pregnancy resulting in a live birth was 13.7 months (11.9 to 15.5) for the intervention and 14.7 months (12.9 to 16.5) for the control group, and respectively 13.0 months (11.3 to 14.7) and 14.0 months (12.2 to 15.7) for the mean time to a clinical pregnancy.

### Methods of conception and fertility treatments

In the intervention group, the rate of natural conception was significantly higher compared to the control group (*P* = .014), with an absolute increase of +21.0%, a 2.7-fold increase and a NNT of 5 (95% CI, 3 to 18). This difference remained significant after adjusting for potential confounders (*P* = .005). Additionally, the intervention group had a 3.3 times higher rate of achieving a clinical pregnancy using IUI, close to statistical significance (*P* = .058), with an absolute increase of +12.3% (95% CI, −0.3% to 24.3%). Notably, there were no significant differences between groups in the use of fertility treatments, including no fertility treatment, or the mean number of cycles per woman as shown in Table 6.

**Table 6:** Methods of conception and outcomes of fertility treatments 18 months after randomization, for women included in the modified per protocol analyses

Outcome	Intervention group (n = 51)	Control group (n = 57)	Risk ratio (95% CI)
**Method of conception leading to clinical pregnancy (percentage of women)**			
Natural	17 (33.3%)	7 (12.3%)	2.71 (1.23 to 6.01)*^a^*
Ovulation induction, without IUI	4 (7.8%)	8 (14.0%)	0.56 (0.18 to 1.75)
Intrauterine insemination	9 (17.6%)	3 (5.3%)	3.35 (0.96 to 11.7)
IVF (with or without ICSI) or FET	1 (2.0%)	3 (5.3%)	0.37 (0.04 to 3.47)
**Fertility treatments (percentage of women)**			
Any fertility treatment	35 (68.6%)	40 (70.2%)	0.98 (0.76 to 1.26)
Ovulation induction	35 (68.6%)	37 (64.9%)	1.06 (0.81 to 1.38)
Intrauterine insemination	21 (41.2%)	17 (29.8%)	1.38 (0.82 to 2.31)
IVF (with or without ICSI) or FET	2 (3.9%)	4 (7.0%)	0.56 (0.11 to 2.92)
**Fertility treatments (number of cycles)**			
Ovulation induction	163	162	N/A
Intrauterine insemination	73	62	N/A
IVF (with or without ICSI) or FET	5	9	N/A
**Cycles per woman*^b^***			
Ovulation induction	2 [0-5]	2 [0-5]	*P* value = .865
Intrauterine insemination	0 [0-2]	0 [0-1]	*P* value = .331
IVF (with or without ICSI) or FET	0 [0-0]	0 [0-0]	*P* value = .448

Results are reported as frequencies (with percentages).

Abbreviations: FET, frozen embryo transfer; ICSI, intracytoplasmic sperm injection; IUI, intrauterine insemination; IVF, in vitro fertilization.

^*a*^P = .005 after correction for smoking status, PCOS status, parity, BMI categories, and male or tubal factor.

^*b*^Non-normally distributed variables are presented as medians [with interquartile range] and *P* values from Mann-Whitney U test.

## Discussion

In this pragmatic randomized controlled trial, a 6-month lifestyle intervention alone and then combined with fertility treatment in women with obesity and infertility showed a nonsignificant increase in live-birth rate (primary outcome) compared with usual fertility care in both the ITT analyses, which included all participants, and the mPP analyses, which were restricted to those who completed at least 6 months. Although this increase did not reach statistical significance, and therefore our primary objective was not met, the absolute increases in the live-birth rate with our intervention of 8.5% (NNT of 12) for ITT and 14.2% (NNT of 7) for mPP may be clinically significant if confirmed. Indeed, these absolute percentage points are aligned with the minimum clinically important differences considered by the experts in our network, namely an absolute difference of 15% using the mPP principles and 10% using ITT principles (34).

The intervention was most effective in improving secondary fertility outcomes, by significantly doubling the rate of natural clinical pregnancy in all women (ITT); and by significantly increasing the clinical pregnancy rate by 58% and nearly tripling the rate of natural clinical pregnancy in participants who had completed at least 6 months (mPP). These important clinical outcomes were achieved even though the proportion of participants who did not perform any fertility treatment and the number of cycles of fertility treatment per woman were the similar between groups, and without differences in miscarriage rates. These analyses remained significant even after correcting for potential baseline confounding variables.

To our knowledge, only 3 published RCTs have reported on the impacts of a lifestyle intervention on fertility outcomes in women with obesity and infertility (21, 22, 35). Sim et al (22) found that women following a very intensive lifestyle intervention (n = 27) vs standard of care (n = 22) for 12 weeks before fertility treatments had a significantly higher 12-month pregnancy rate (48% vs 14%, *P =* .007). In this trial, both groups did not start fertility treatment until 12 weeks, in contrast to our pragmatic RCT in which the control group started fertility treatment immediately, which favors the control group. Another recent RCT compared an intensive pharmacologic plus lifestyle intervention to a weight-neutral exercise intervention during 16 weeks before fertility treatments (35). This study found that acute weight loss before fertility treatments (n = 191) did not improve fertility outcomes after 3 cycles compared with exercise without weight loss before fertility treatments (n = 188). This trial used an intensive weight loss intervention compared to a control group that received a lifestyle intervention, not usual care, such that results are difficult to compare with ours.

A large multicenter RCT published in 2016 (21) assessed a lifestyle intervention similar to ours that was provided alone for 6 months and then stopped when fertility treatments were initiated, compared with prompt initiation of fertility treatments. They reported that women who were randomized to the lifestyle intervention (n = 280) had a significantly lower 24-month live-birth rate compared with control women (n = 284) (43.9% vs 53.9%) and a similar clinical pregnancy rate (62.5% vs 65.5%), but a significantly higher rate of natural pregnancy (26.1% vs 16.2%). The 6-month between-group difference in weight loss was comparable to our RCT (−3.3 kg vs −2.7 kg in our RCT), but their lifestyle intervention was delivered by a nurse or dietitian for only 6 months, whereas our program was delivered by an interdisciplinary team of professionals (dietitian and kinesiologist) for the entire duration of the study, including during fertility treatments. Our population was more obese (BMI = 39.7 vs 36.0 kg/m^2^), more often multiparous (40.2% vs 23.2%), and had a higher proportion of anovulatory infertility (76.4% vs 46.9%). Another important difference is that in the Mutsaerts RCT, the percentage of women who underwent any fertility treatment was significantly lower in the intervention group than in the control group (63.2% vs 81.3%), whereas in our RCT it was comparable between groups (66.7% vs 64.1%). As mentioned in the “Methods” section (regarding the interventions), it is not surprising that participants in each group received the same intensity of fertility treatments even if the intervention group started their treatments 6 months later, as most of them would complete all their fertility treatments within 12 months (within a study follow-up of 18 months for both groups).

One important concern for women is that postponing fertility treatments for 6 months to follow a lifestyle intervention might delay or reduce their chances of conceiving and giving birth. However, our results show that after 18 months, pregnancy and live-birth rates were actually higher—though not significantly—in the lifestyle intervention group than in controls, with similar use of fertility treatments. This suggests that delaying fertility treatments to allow the lifestyle intervention to work does not lower overall birth rates, and it does increase natural pregnancies. Additionally, Fig. 2 shows overlapping pregnancy curves in both groups during the first 6 months, with the control group even having slightly fewer clinical pregnancies (Fig. 2C). This indicates that pregnancy is not delayed by postponing fertility treatments in women undergoing such lifestyle intervention.

Our lifestyle intervention may improve fertility in women with obesity and infertility primarily by reducing adiposity (mainly central adiposity), which can improve hormonal imbalances, insulin resistance, adipokines, lipotoxicity (36), inflammation, mitochondrial dysfunction, and others (17, 37-39). They may also benefit women with obesity without PCOS. Future research should explore these mechanisms further.

### Strengths and limitations

An important strength of our study was the duration of the lifestyle intervention provided to the participants, which continued in combination with fertility treatments for a maximum of 18 months. Moreover, the quality of the intervention was ensured by professional counseling from both a kinesiologist for physical activity and a dietitian for nutrition, who were trained in motivational interviewing.

One limitation of our study is the lack of statistical power to definitively conclude differences in live-birth rates between the groups, which might indeed be clinically significant if proven accurate (34). However, there are still few published studies using live-birth rate as the primary outcome, and our study had sufficient power to detect clinically important secondary outcomes, such as clinical pregnancy rate and natural pregnancy rate. Our trial may have recruited women who were more motivated or obese than the usual population of women with obesity who consult fertility clinics. This is not a significant limitation in a pragmatic RCT such as ours, because in real-life situations, women with obesity consulting at a fertility clinic would need to be motivated to engage in some sort of lifestyle program. This aspect of the trial actually increases the external validity of our results.

The inability to blind the intervention allocation could have introduced biases, but the research protocol was pragmatic and reflected real life. The fertility outcomes were also objective. Our mPP analyses are subject to nonrandom attrition, so the loss of more participants who were not adherent in the intervention group may have led to an overestimation of treatment effects. However, this also reflects real life, and these analyses are important to answer the most clinically important question for participants, which is whether the intervention is effective in improving their fertility if they complete the first 6 months of the lifestyle intervention, during which they postpone fertility treatment. Importantly, the drop-out rate in our mPP analysis (15%) was lower than the median drop-out rate of 24% reported in a systematic review of lifestyle programs for this population (median follow-up of 6 months) (40).

## Conclusion

A 6-month lifestyle intervention alone, subsequently combined with fertility treatments if required, resulted in a nonsignificant increase in the 18-month rate of pregnancies leading to a live birth (primary outcome) and a significant increase in the rate of natural pregnancies, compared to usual fertility care from the onset. Among participants who remained in the trial for at least 6 months, this lifestyle intervention improved significantly the 18-month clinical pregnancy rate, especially for natural pregnancies. These results were achieved even though the same proportion of participants in both groups did not receive fertility treatment (by ITT and mPP). Since the primary outcome of this trial was not met, more research is needed in a multicenter RCT setting and with appropriate statistical power.

## Data Availability

Restrictions apply to the availability of most data generated during this study to preserve patient confidentiality. The corresponding author will on request detail the restrictions and any conditions under which access to some data may be provided.

## Clinical Trial Information

ClinicalTrials.gov registration: NCT01483612

## Abbreviations

BMI: body mass index
IUI: intrauterine insemination
IVF: in vitro fertilization
mPP: modified per protocol
NNT: number needed to treat
PCOS: polycystic ovarian syndrome
RCT: randomized controlled trial
